# Bystander Programs: Accommodating or Derailing Sexism?

**DOI:** 10.3390/bs7040065

**Published:** 2017-09-27

**Authors:** Adam Reid, Lauren Dundes

**Affiliations:** 1 Department of Campus Safety, McDaniel College, Westminster, MD 21157, USA; 2 Department of Sociology, McDaniel College, Westminster, MD 21157, USA

**Keywords:** bystander, sexual assault, college students, sexism, intoxication, prevention, gender roles

## Abstract

Bystander programs implemented to meet federal requirements to reduce sexual assaults on college campuses in the United States must include primary prevention. Survey data (*n* = 280) and interview data (*n* = 20) presented in this paper explore students’ hypothetical and actual willingness to intervene as bystanders. Although most students surveyed (57%) claim they would be very likely to intervene, fewer than half would be very suspicious of someone leading away an intoxicated individual at a party (45% of women and 28% of men: *p* < 0.01). Interview data reveal how students perceive risk factors at college parties and what types of bystander measures they attempt, including “distractions”, a nonconfrontational tactic in which bystanders avoid more direct but socially risky interventions. Subsumed in many current bystander programs is an invisible element of valorizing harmony. Condoning bystanders’ unwillingness to directly confront seemingly predatory individuals could make change seem out of reach and could also embolden offenders whose behavior is observed and only temporarily thwarted.

## 1. Introduction

Colleges and universities across the United States are working to reduce campus sexual assaults in accordance with the mandates of the Violence Against Women Reauthorization Act of 2013 and the Campus SaVE Act [1,2]. Mandated training requires some type of bystander intervention that encourages students to monitor and intervene in their peers’ behavior that includes dating violence and sexual assault [3]. This federal mandate is extremely broad, encompassing both sexual assault awareness and prevention efforts, all of which is fluid, subject to changing political forces [4,5]. Programs differ in the type of bystander behavior encouraged but often include a variety of interventions, e.g., checking on friends at parties, telling friends they do not have to tolerate violence from partners, asking friends who seem upset if everything is okay or if they need any help, asking friends periodically about how things are going in their relationship, and trying to remove friends from an uncomfortable situation (such as asking, “Would you come to the bathroom with me?”) [6].

The newness of bystander programs raises a host of issues, from the broadness of the goals to clashes with the right to autonomy. When colleges portray bystander intervention as a moral duty in defense of the “powerless”, students may not understand that legally there is no affirmative duty for them to intervene, a facet usually omitted from bystander training. In general, students who fail to act as bystanders are not responsible for the risk created by others nor the harm that may ensue, even if a danger is foreseeable. Because bystanders do not receive legal protections if they choose to fulfill a moral duty [7], bystander programs should receive critical assessment, regardless of noble intentions to reduce trauma due to sexual assault. 

This paper examines issues surrounding the primary prevention of sexual assaults, with a specific focus on strategies to protect women at college parties who are at risk of sexual violence. Central to these efforts is bystanders’ ability to discern which interactions merit intervention. Bystander action relies on awareness and correct interpretation of a number of cues, many of which are nonverbal, an ambiguity complicated by student drinking [8]. Moderating alcohol use, however, remains the elephant in the room, conspicuously absent from many bystander programs and their evaluation [9], despite its importance in designing and implementing bystander programs [10,11,12]. For example, binge drinking may depress empathy, a possible risk factor for objectifying and depersonalizing women [13]. In addition, bystanders tend to have less prosocial attitudes when they drink [14]. Moreover, alcohol contributes to misreading consent to hook up [15,16], as well as hookup regret [17]. 

To add to the dialogue about the challenges of bystander programs in colleges, we present survey data showing the extent of students’ hypothetical willingness to intervene to prevent sexual assault. We also share interview data to reveal how potential student bystanders read these cues and factors that affect whether they would intervene in situations they have observed in which primary prevention appears to be an option. 

These results are linked to intense social pressures prevalent at college. In particular, the risks posed at college parties are key to understanding the norms and context that perpetuate the devaluing of women. We discuss how these psychosocial factors are relevant to program design, as exemplified by “distractions”—a tactic in a popular program, Green Dot—that arguably gives license to bystanders to skirt more direct but socially risky interventions. 

## 2. Green Dot

The Green Dot program merits scrutiny due to its popularity and “promise” [18] as a means to curb sexual violence. Despite limitations of data supporting its effectiveness (e.g., a participant response rate under 50% [19]), the Green Dot program is a widely used means for colleges and universities to satisfy the need for “safe and positive” options for bystander intervention to “prevent harm or intervene” in risky situations, mandated by the Violence Against Women Reauthorization Act that amended the Clery Act [20]. Over 100 colleges and universities have used Green Dot, including the University of California, Berkeley, the University of North Carolina, Chapel Hill, and Harvard University [21]. Green Dot teaches program participants how to intervene in situations that warrant on-the-spot involvement. The goal is to change a campus’ culture by providing the tools to become an effective bystander who is both reactive and proactive in checking power-based personal violence. Active bystander behaviors are counted as green dots to distinguish them from red dots that signify behaviors associated with violence [22]. 

Green Dot bystander education can take many different forms, from a Green Dot speaker on a single occasion to full Green Dot training, the latter of which seems to be more effective [23]. A centerpiece of Green Dot is its panoply of options for stepping in, divided into three “D’s”: direct action, delegating to (i.e., calling on) others (like campus safety), and distractions. Distractions are a way for conflict-averse individuals unable to stand up to social pressure when they see what they perceive as inappropriate behavior. Specific examples of Green Dot distractions include purposefully spilling a drink and starting to juggle, while specific advice includes the following: (1) If bystanders overhear statements ridiculing, demeaning, or belittling others, they can change the subject or introduce a new topic; (2) Bystanders who observe aggressive or threatening physical contact or body language can distract the threatening individual by asking him for directions or about the previous night’s soccer game; (3) Those who overhear stories about sexual escapades that “do not sound entirely consensual” could cause a scene, drop something, or stumble and fall to redirect attention to themselves as bystanders [22]. 

## 3. Challenges of Operationalizing Bystander Action

While there are benefits to programs like Green Dot that allow students to be active bystanders according to their individual comfort level, the broadness of program goals coupled with flaws in measuring instruments can obfuscate how much colleges are actually accomplishing to reduce sexual assault by adding bystander programs to their armamentarium. The rush to execute these programs has led not only to condoning significant program flaws, but also to a willingness to forego baseline knowledge needed to determine the impact of bystander programs on sexual assaults. In addition, many bystander programs encourage intervention without identifying and parsing the full range of opportunities to intervene, including primary, secondary and tertiary measures [24]. 

The complexity of measuring the extent of the problem has contributed to data of varying quality and usefulness. For example, a wide variety of behaviors may be considered to be relationship violence. In some cases, child abuse, bullying, and fighting among friends or siblings (as young as age four), are included, as well as emotional violence, defined broadly (e.g., being put down or made to feel bad about oneself) [25]. Other suggested bystander actions lack a clear connection to sexual assault: e.g., I speak up against racist jokes [26]. These strategies have social value but limited applicability to efforts to reduce sexual assaults in college. 

Many of the outcome measures are responses to hypothetical scenarios (vignettes) that equate respondent willingness to intervene with confidence in being a bystander [27,28,29]. Even confident bystanders, however, still may not be able to recognize the often-subtle cues signaling when intervention is needed, assuming they are observant or sober, which is especially important in settings that are often dark and loud. Nor can we assume a high correlation between how respondents claim they would react in a hypothetical scenario and what they would do in the heat of the moment [30]. 

Some of the questions describe unambiguous scenarios in which a woman is clearly in distress, suggesting a “right” answer (see [31]). In reality, however, it can be difficult to discern when intervention is warranted—even for survivors. According to one analysis of why survivors do not report offenses to university officials, the most commonly mentioned reason (cited by 30%) was, “It was not a big enough deal” [32]. Social desirability also could affect responses since most respondents know the morally correct answers. Scenarios with clear “right” answers ignore that most stories have at least two sides, accounting for the great controversy surrounding many incidents. We gain little by asking students if they would respond to scenarios where intervention is clearly warranted. For example, Moynihan et al. [33] present the scenario of a woman who is “uncomfortable”, ignoring that this determination of a person’s mental state is one of the challenges of bystander intervention. Furthermore, egregious, indisputably wrong behavior, such as that of Stanford University student Brock Turner [34], is not the norm. 

Questions that deal with bystander primary prevention intervention are limited in number and often included as part of composite scales with a continuum of factors that does not pinpoint whether bystander behavior is “showing displeasure” at a sexist comment or actually intervening in a situation that seems to be risky, like a scenario in which a drunk person is being led forcefully from a party to a residence hall room [26,35,36,37]. The broad range of targeted behaviors obscures the success of sexual assault prevention. In addition, bystander behaviors assessed may be specific to friends who elicit greater empathy [27,30,38] with unknown generalizability to acquaintances or strangers. 

When individuals intervene with potential offenders who are not friends, we can expect reliance on distraction tactics (rather than direct confrontation), partly because of a cultural preference for harmony over justice [39,40]; this leaves aggression unchallenged, and undermines efforts to decrease sexual assaults. On the other hand, more direct bystander interventions pose often-unrecognized implications for legal liability and safety, making distractions a more attractive option. Regardless of whether intervention is direct or subtle, the broad array of actions that qualify as compliance may result in a threshold too low to translate into improved outcomes for women. Nevertheless, the requirement to comply with federal bystander mandates need not be linked to any particular outcome. According to Culture of Respect (an initiative of Student Affairs Administrators in Higher Education [NASPA]), evidence for sexual assault prevention efforts qualifies as “emerging” when “there is an expected effect of [a] program because it is based off sound theory and previous research. This might mean that there is evidence that participants and administrators are satisfied, but no evidence that learning objectives were achieved” [41]. This level of “evidence” (albeit the minimal level acceptable) reflects a need for more studies and critical analysis of sexual assault prevention. This study examines how sociological factors can inform bystander program design to increase the probability of avoiding feel-good measures that satisfy compliance requirements in favor of those more likely to make students safer.

## 4. Methods

In accordance with purported benefits of student involvement in the study of sexual assault [42], this paper involves student and faculty collaboration. Quantitative survey data examined in this paper were collected in fall 2015, as part of a required methods course in sociology at a small, mid-Atlantic liberal arts college with about 1700 undergraduate students. Two-hundred and eighty (280) undergraduate students (55% of whom were female) comprised a systematic random sample of college students that completed a five-page survey (67% response rate). The class survey included a variety of approximately 30 variables (exclusive of demographic information) such as the Rosenberg self-esteem scale, body image, attitudes about alcohol, and perceptions of social mobility. The set of survey questions examined in this paper assessed hypothetical bystander behavior (see Table 1) and whether being suspicious of someone leading away an intoxicated person from a party correlated with perceptions that alcohol is more helpful than harmful in social situations (see Table 2).

The entire student population was part of the sampling frame that excluded only commuter students (because data were collected from students living in residence halls). Students collecting data were required to return up to three times to a residence hall room assigned to them to solicit participation from one resident in each designated room. After students agreed to take the survey, they completed a consent form that was returned to the student researcher. The respondents then filled out the survey, while the student researcher waited a short distance away. Respondents placed completed surveys in an envelope that they sealed and then placed inside a larger envelope to assure anonymity. Survey respondents were comparable demographically to the entire campus population, with the exception of an overrepresentation of first year students who responded (37% of respondents were first year students compared to 26% of the overall campus population). 

These quantitative data were supplemented with qualitative data collected in fall 2016 as an independent project, when one of the co-authors, a student in the fall 2015 Methods class who was also employed as a Campus Safety officer (then 29 year old), recruited 20 participants exposed to the Green Dot program through opportunity or snowball sampling. The co-author disclosed his employment as a Campus Safety officer while indicating that the research was conducted apart from any employment duties, outside of work hours, as faculty-supervised independent research about bystander behavior at parties. Five out of 25 students (20%) students approached declined to participate, noting that they did not attend parties. Interviewees included 11 female and 9 male volunteers, from several different majors: Sociology (5), Exercise Science (4), Economics (2), English (2), History (1), Psychology (1), Political Science (1), while some (4) were undecided. Respondents were comprised of 9 men, all of whom were white except for one Black student and one Asian student in addition to 10 white women and 1 Black woman. These individuals were interviewed individually in a private space by the student Campus Safety officer co-author as well as a female professor of sociology (in her fifties). Prior to the start of the interviews, all interviewees were informed both in writing (in the consent forms) and verbally that both interviewers were mandatory reporters required to report any incidents covered by Title IX or the Clery Act that were disclosed to them. Interviews lasted between 10 and 30 minutes.

The Institutional Review Board approved interview questions were inspired by the HEDS Sexual Assault Campus Climate Survey [43]. Questions were open-ended, focusing on whether interviewees had seen suspicious activity that might lead to sexual assault, whether they intervened and reasons for their intervention or decision not to do so. The conversations incorporated fixed questions but were free flowing and documented with handwritten notes. Responses were not tape recorded in order to increase interviewee comfort given the sensitivity of the topic. All respondents signed an informed consent form that apprised them of the confidential nature of the data as well as resources for those who had been traumatized by sexual assault or relationship violence. 

## 5. Results of Survey Data

Survey respondents were asked the following: How likely are you to (1) be suspicious of someone you see leading away an intoxicated individual at a party? (2) Intervene if you witness suspected unwanted sexual contact; (3) Call Campus Safety or a resident assistant if you suspect someone is a victim of unwanted sexual contact? (See Table 1)

There is a gap between students being very likely to be suspicious of someone leading away an intoxicated person from a party (37%) and their stated willingness to be very likely to intervene (57%). As a result, the extent that students actually intervene could be limited by not tapping into cues that a risky situation is afoot that at least warrants suspicion (aside from whether intervention might be needed). This gap is most notable for men who were much less apt to indicate that they were very likely to find a situation suspicious (*p* < 0.01). It is notable that a statistically significant gender difference exists for those very likely to be suspicious, a possible reflection of women’s greater awareness of circumstances that put them at risk of sexual assault. 

We also examined how attitudes about alcohol predicted the above-mentioned suspicion variable. An 11-item scale (Cronbach’s alpha: 0.793) included items such as attitudes about whether alcohol enhances social activity, facilitates connections with peers, facilitates same-sex bonding, allows people to have more fun, causes students to miss or fall behind in class, and increases reckless activity. Those with a more positive attitude about alcohol were less likely to be suspicious of someone leading away an intoxicated person from a party although the correlation between these attitudes and willingness to intervene or call for help was not significant (see Table 2).

## 6. Interview Data

### 6.1. Types of Scenarios Witnessed

Most (14/20 or 70%) of interviewees had seen a suspicious situation, ranging from 1 to 3 incidents. All of these situations reportedly prompted interviewee action, which entailed interrupting the suspicious interaction but without direct criticism of the males who appeared to be overstepping, except in one case. The sole person who bluntly chided a peer was a tall male athlete with a large build (and military training) who directly confronted a friend he thought was overly aggressive toward a woman. Even this respondent, however, emphasized that his intervention was executed in a “joking” manner in which he told his friend to stop acting “rapey”. 

The respondents that intervened consistently first kept a close eye on the person who appeared to be at risk, as they waited and watched to see if the situation escalated. This kind of surveillance was deemed necessary because of the risks posed by alcohol (a contributing factor that all 20 students brought up on their own, regardless of whether they had ever witnessed a suspicious situation). 

One male recounted: “I hear stories all the time about women hooking up when they don’t want to because of drinking, and regretting what happened. It’s not like girls say they’ve been raped, but that they’ve made ‘mistakes’. This is what some of the older girls hear from the younger ones who come to them for help and advice”. 

A female commented: “I know to look out for my friends when I see them intoxicated and not in a condition to make a good decision, or they have a guy all over them, touching them. If a guy is hanging around, sticking around waiting for one of my friends, in any of these situations, I’ll grab some of my friends to help me lead her away from the guy or walk her back home”.

Seven students had observed the following scenario that they thought required intervention: a male student offers to take an inebriated female student back to her residence hall or apartment, even if he barely knows her. In order to make this assessment (whether individuals knew each other), however, interviewees were best able to judge whether their friends (rather than strangers or acquaintances) were at risk. Their bystander tactics included interrupting the conversation and physically steering their friends away to another part of the room, or taking them back to their residence hall rooms if they seemed especially intoxicated. 

### 6.2. Risk Factors Seen as Cues Warranting Intervention

Given the difficulty of offering specific guidance to students about when to engage in direct bystander intervention, we were especially interested in how students identified risk factors that would cue intervention. According to 10 interviewees, warning signs of when to intervene included one or more of the following: unsolicited and unwanted attention, when a man comes on too strong without any signals of approval or encouragement from a woman, when a man violates the personal space of a woman by touching her shoulder, thigh, or buttocks, especially when he does so abruptly, without any preamble (small talk). 

Five female interviewees mentioned that at parties, women they knew tried to get the attention of their friends and communicate with their facial expression and body language that they wanted to extricate themselves from an interaction. They corroborated the existence of social norms discouraging outright rejection of a sexual aggressor. 

A female interviewee commented: “You may not want to outright say no, especially if you are manipulated because of an emotional attachment. People want to be seen as a social insider”. 

Three interviewees paid particular attention to men who had been turned away by multiple women then searched for any woman open to their advances. One female interviewee shared that she intervenes on behalf of her friends approached by “a guy who has made multiple attempts to get with a girl, including dancing with lots of different girls, as if indiscriminately looking for a sex partner”. 

Male peer approval was commonly cited as putting women at risk of sexual assault. Eleven interviewees stated that males who take advantage of women who have had too much to drink were generally candid with their friends about their intentions to find a sexual partner, part of a cultural milieu that encourages men to hook up in order to impress peers. One female respondent said, “I try to intervene if I have any sense that the guy is insincere in his interest and just trying to impress his peers”. Although both male and female students noted these patterns, one male noted that, “There is no social capital when males confront other males, and call out their actions. Women do respect guys sticking up for them but men risk being ostracized”. This male’s concern about social exclusion extended to acknowledgement of men’s status earned by having a large build, as he stated that he would be more inclined to confront a few smaller men as opposed to one big individual, a manifestation of how masculinity conflates with bystander action. 

While males had to contend with pressure not to impede other men’s sexual conquests, women were more concerned with protecting their friends’ reputations. When we inquired how interviewees knew when to intervene in the absence of cues from a friend who might be intoxicated, two women said that they took the liberty of intervening when they thought that if sober, their friend would not want to be sexually involved with that person, a decision they admitted was based largely on physical appearance. Both expressed that a lack of selectivity could hurt their friends’ reputation, especially with repeated hookup “infractions”, given the tendency for gossip on a small campus. 

One female interviewee explained both phenomena:

“I look out for my friends and watch whether the guy is checking for affirmation from his friends. They are very aware of whether their peers are watching, if they are being observed”. In reference to friends’ inebriation, she said: 

“I try to think about if my friend would regret it,” which she clarified (when asked) is determined by her assumption that her friends would only hook up with “someone attractive with a good personality. You want to protect your friends’ rep, to keep people from talking about it.”

Respondents also indicated circumstances that caused reluctance to intervene. Three mentioned hesitance when the aggressor was with a group, especially when it was comprised of a sports team or fraternity. This was particularly important if the respondent had to resort to acting alone as a bystander, without backup. Five respondents indicated more comfort with intervention when they succeeded in convincing others to help them when they intervened. 

### 6.3. The Role of Bystander Education

While 14 interviewees mentioned the importance of bystander education, four said they did not feel that they had received sufficient guidance on how to be a bystander and only remembered that they had received training, but not specific details. One male (a junior) recalled receiving the “Green Dot” training in his freshman year but remembered just that the program was about “looking out for people”. A female student praised the bystander program but admitted that she did not learn anything that would change her own behavior. Instead, she said she felt reassured knowing that others had been exposed to the program. 

## 7. Discussion

This research specifically focused on bystander behavior at campus parties, and not relationship violence or other types of bystander behavior (e.g., condemning racist jokes). In addition, although we adopt a hetero-bias in our discussion, most incidents warranting bystander intervention appear to involve heterosexual interactions, as suggested by one study in which 90% of aggressive bar room incidents involved male initiators with female targets [44]. 

Our quantitative data indicate that students are willing to be bystanders: 90% of students are either somewhat or very likely to intervene if they “witness suspected unwanted sexual contact” with 57% identifying themselves as “very likely” to intervene, suggesting that the majority of students do not need to be convinced to act in this role, at least in response to a hypothetical situation. Most also would be willing to call for help if they suspected someone was a victim of unwanted sexual contact. 

Nevertheless, respondents, especially males, were less likely to be suspicious of someone leading away an intoxicated individual from a party, perhaps because of the vagueness of the scenario (which is addressed to some degree in our interview data). Despite the limitations of the question, our finding that only 37% would be very suspicious suggests that programmatic efforts to urge students to intervene should be refocused on educating students on how to determine when intervention might be warranted. The gender gap in those who would be very suspicious—28% of males versus 45% of females—implies that women are more in tune with risk factors for sexual assault while males may unconsciously desire to downplay male culpability (especially if their own behavior has ever matched what was described). We also found evidence that those who see alcohol as having a positive impact on social life were less apt to be very suspicious of someone leading away an intoxicated person at a party, indicating that education about alcohol, including how it impairs the ability to consent (see [45]), should be incorporated into all bystander programs. 

Our interview data also reveal the tendency of women to resort to subtle ways of extricating themselves from uncomfortable situations, consistent with gender norms that contribute to condoning male aggression and discouraging female assertiveness. None of our interviewees questioned the need for women to signal for help because they wanted to avoid directly rejecting a man. Laura Nader [39,40] proposes that we live in a society where we cannot speak our minds, where we prioritize harmony over justice. So for example, if a man is inappropriately putting his hands on a woman, violating her personal space or hovering, a woman may be hesitant to appear “impolite” by directly telling him to stop. None of the interviewees disputed this cultural preference for harmony over justice. The role of bystander intervention in helping women rid themselves of unwanted male attention hinges on an understanding that women face the delicate task of indirectly rejecting a man’s advances, and maintaining an identity as nurturing and not ego crushing. 

Deborah Tannen details the many ways in which women, often unconsciously, fulfill gender-based expectations in their interactions. Her work explores how women who have too many masculine traits like directness, confidence, certainty and a take-charge attitude are deemed unfeminine and unlikeable. Too many feminine traits like being apologetic, hedging, showing great concern for the feelings of others and helping them save face, make them appear weak and unsure of themselves [46,47]. The cultural prescription of female indirectness is particularly problematic, especially when men are under the influence not just of alcohol, but pressures to succeed in their sexual conquests [13]. 

The culture of objectifying women took little prodding to unearth. Although both males and females admitted that a major criterion for hooking up was superficial, based on physical attractiveness, a male in our study confessed that the ranking system (1–10) was still a popular means of assessing women, a tool also revealed to be part of a “sexual scouting report” on the Harvard women’s incoming soccer players. The ten-point rating scale had been employed long term by the Harvard men’s soccer team, resulting in Harvard athletic director Robert Scalise cancelling the remainder of the men’s season in fall 2016. As the Harvard women who were demeaned reported, “We have seen the ‘scouting report’ in its entirety. We know the fullest extent of its contents: the descriptions of our bodies, the numbers we were each assigned, and the comparison to each other and recruits in classes before us” [48]. 

Status from hookups relates to the attractiveness of partners, a fact also reflected in the term “beer goggles”, meaning that alcohol consumption makes an unattractive potential mate appear more appealing. More men use this term (that perhaps partly reflects that the consumption of beer is more associated with males than females) [49]. One of our male interviewees talked about how males give each other flak when one of their peers hook up with someone deemed unattractive, even though some status is accorded for “scoring” (a factor corroborated by research on the impact of the perception of peers’ approval of sexual behavior, including sexual aggression [14]). In comparison, women who hook up with someone deemed undesirable can be stigmatized, and sometimes traumatized (if coercion was involved), without any of the ambivalence that occurs when males have sex with partners deemed subpar. This double standard is exemplified in the applicability of the “walk of shame” only to women [50]. These criteria make problems rooted in sexism even more difficult to combat, especially given the confluence of alcohol, attractiveness standards, and peer pressure. 

Three of our male respondents revealed that they thought that it is normative for men to make comments about women’s sexual attractiveness, and that talking about women in this manner does not necessarily constitute actively disrespecting women. With some embarrassment (and disavowal of support for Donald Trump), one male student referenced Trump’s infamous “locker room” talk as typical. Two interviewees noted the significant peer pressure, especially for males, to hook up, and to avoid “cock blocking” or sabotaging other males’ attempts to hook up, especially the opportunity to gain status from an attractive partner [51]. Similarly, the term sausage fest denotes a party that is unsuccessful because of the predominance of men and a paucity of women (presumed to offer unfavorable odds for men to hook up). Aside from corroborating that males gain status through sex, the term also reduces the identity of men to phalluses, a parallel of the term jock for male athletes [52]. There is no equivalent term when the gender ratio is reversed, suggesting that women have different priorities in attending parties. 

The objectification of women is well known in such crass terms as “paper bag” or “brown bag” sex, indicating that a woman’s body but not her face is sufficiently attractive. In other cases, the targets of men’s sexual pursuits are overweight women perceived as vulnerable, a phenomenon called “hogging” or “whaling”. This has been deemed a homosocial competition reflecting men’s attempts to bond with and impress their peers achieved with tales of their conquests or betting about the size of the women they can attract [53]. This type of behavior is of particular concern for those interested in bystander programs, as women who are deemed less worthy are also less apt to have others intervene on their behalf [54]. On top of commonplace degrading comments and condoning men’s sexual conquests is a culture proscribing snitching. Our students’ observations about consequences for men who step in to stop other men from badgering women or cajoling them into sex are generalizable [55]. Partly as a result of these social pressures, bystander programs offer a less direct method for intervening: distractions. 

Encouraging bystander behaviors that are nonconfrontational, e.g., spilling a drink on someone, prioritizes harmony, and treats it as the natural order of interactions [40]. By placing distractions, a harmonious resolution, on the same level as direct action, we lose an opportunity to challenge male hegemony. Instead, we slow social change when we sanction bystander intervention that deflects, de-escalates and delays in a particular situation, but does not challenge sexism. 

Although males’ efforts to impress other males in an effort to avoid any signs of homosexuality are a strong social force driving hookups [55], these pressures should not be considered insurmountable, but instead should be taught as part of efforts to explain the need for direct bystander intervention. If men are conscious of peer expectations to demonstrate unambiguous heterosexuality, then this topic could be incorporated into bystander training, as part of urging direct intervention that is needed to combat these strong social pressures to prove masculinity [55,56]. Even in the short term (setting aside more lofty, long-term goals of fighting sexism), if bystanders are more assertive in directly condemning the behavior, these “direct” actions could not only help deter some men from perpetrating unwanted actions, but also could give surrounding bystanders more cues as to the appropriateness of intervening, and expedite normalizing this kind of community response. In implementing bystander programs, however, we must avoid implying that women cannot fend for themselves, which could lessen the likelihood that they directly speak up for themselves assertively. Furthermore, too much focus on bystanders could detract from individuals’ own efforts, reinforcing what Nader calls trustanoia, the opposite of paranoia: trusting others to protect one’s interests [57]. 

We must also take into account that bystanders are supposed to play a role in protecting women, even in the absence of knowing quite what to do [58]. In assessments of bystander behavior, the broad range of outcome measures shows that we cannot pinpoint what we are looking for. Programs encompass everything from direct intervention in a clearly risky situation to checking people who are sexist, racist and homophobic. While a multipronged effort is indeed warranted, the problem is that for primary prevention (as opposed to reporting or comforting a survivor after the fact), it is very difficult to know when intervention is warranted as well as the potential legal consequences. College students are adults who should not be infantilized yet we also want to instill the benefits of membership in a community. 

The distraction bystander options tacitly reinforce that women have limited choices if they want to be perceived as feminine when they intervene. These less direct options accommodate invisible social rules that mark women as undesirable for violating feminine norms for civility and conflict aversion [46,47] and expectations for harmonious interactions. On their web site, Green Dot quotes Paulo Freire: “Washing one’s hands of the conflict between the powerful and the powerless means to side with the powerful, not to be neutral” [59]. Not only does this quotation within the context of bystander intervention portray vulnerable women as “powerless”, but it also encourages bystanders who employ distractions to feel like they are fighting the system. 

## 8. Methodological Concerns

### 8.1. Limitations

The quantitative data presented in this study were a result of a systematic, random sample in residence halls from only one campus that is a small, private mid-Atlantic college. The qualitative data were not representative of the campus as a whole and had the additional limitation of selection bias; the sample consisted of students willing to speak to a fellow student who was employed as a male campus safety officer as well as a female faculty member. Social desirability may well be reflected in the finding that all of the students interviewed reported that their observance of a situation in which someone was at risk had resulted in some type of bystander action (albeit indirect). Respondents also may have omitted information they thought could trigger a formal report. In addition, in this paper, we consider suspicion about someone leading away an intoxicated person at a party as a risk factor for the occurrence of sexual assault in the absence of specific data about how often this behavior leads to a sexual offense. 

### 8.2. Ethical Issues

The comfort level of the interviewees was likely influenced by having mandated reporters conduct the interviews. Some students, especially on a small campus, may have felt uncomfortable when asked to share their bystander experiences given the potential for triggering a mandatory report without anonymity. Despite the seeming lack of discomfort among our interviewees, we remained cognizant of our need to be vigilant about signs of student distress as well as the importance of serving as a bridge to forms of support (i.e., informing verbally and in writing all students of resources available to those seeking confidentiality or help due to concerns related to sexual violence and other issues covered under Title IX or the Clery Act). 

## 9. Conclusions

The federal mandate to implement bystander programs requires colleges and universities to grapple with uncertainty about the circumstances that merit bystander intervention and how students should intervene. In response to the need for greater understanding about the effectiveness of these programs in preventing sexual violence, we share data that suggest that students are willing to intervene as bystanders, but avoid techniques that are direct. They also may be unaware of which situations warrant suspicion and how to behave within a social context complicated by traditional gender roles. 

Bystander intervention is generally preferable to inaction, even in ambiguous situations in which the probability of an imminent sexual assault is hard to assess. This thorny reality surrounding bystander action should be acknowledged and discussed. In addition, potential social repercussions of bystander programs that advocate “distractions” require analysis. The range of bystander options recommended allows those intervening to avoid direct confrontations by prioritizing outward harmony over safety. This leaves social forces like the sexual conquest mentality largely unchecked. Accepting bystanders’ unwillingness to directly confront seemingly predatory individuals also could make change seem out of reach and embolden offenders whose behavior is observed but only temporarily thwarted by a distraction. If bystander programs seem to condone the status quo, progress will be elusive. 

The problem of sexual assault must be framed as a community issue, one that enlists men as allies without vilifying them, but which also does not portray women as victims and men as their saviors who rescue them from would-be perpetrators. It is a delicate balance to encourage direct intervention with offenders without reinforcing the paternalistic notion that women are passive and need protection.

Programs must evolve as we learn more about the effectiveness of different types of intervention in various settings. Institutions should continue to assess how they define both rape (such as how to weigh intoxication) and how best to promulgate their particular definition of consent (such as affirmative verbal consent)—factors that must be considered in light of psychosocial influences on college campuses. While mandated bystander programs can provide some peace of mind to a campus community, we must consider whether current efforts are the best use of limited resources to address a critically important problem.

## Figures and Tables

**Table 1:** Likelihood of Bystander Reactions in Three Situations.

	To Be Suspicious	To Intervene	To Call for Help
Not at all likely	13%	10%	12%
Somewhat likely	50%	33%	41%
Very likely *	37%	57%	47%
	100%	100%	100%
* Very likely: male/female	28%/45% *p* < 0.01	54%/59% NS	54%/59% NS

**Table 2:** Suspicion about someone leading away an intoxicated person from a party & Attitudes about alcohol.

	Negative Attitudes about Alcohol	Positive Attitudes about Alcohol
Not suspicious	7%	20%
Somewhat suspicious	49%	56%
Very suspicious	44%	24%

*p*-value = 0.005.
